# Intestinal parasitic infection alters bone marrow derived dendritic cell inflammatory cytokine production in response to bacterial endotoxin in a diet-dependent manner

**DOI:** 10.1371/journal.pntd.0007515

**Published:** 2019-07-01

**Authors:** Stacey L. Burgess, Akihiko Oka, Bo Liu, David T. Bolick, David Noah Oakland, Richard L. Guerrant, Luther Bartelt

**Affiliations:** 1 Division of Infectious Diseases and International Health, Department of Medicine, University of Virginia, Charlottesville, Virginia, United States of America; 2 Center for Gastrointestinal Biology and Disease and the Departments of Medicine, University of North Carolina at Chapel Hill, Chapel Hill, North Carolina, United States of America; 3 Division of Infectious Diseases, Department of Medicine, University of North Carolina, Chapel Hill, North Carolina, United States of America; PUCRS, BRAZIL

## Abstract

*Giardia lamblia* is a common intestinal parasitic infection that although often acutely asymptomatic, is associated with debilitating chronic intestinal and extra-intestinal sequelae. In previously healthy adults, a primary sporadic *Giardia* infection can lead to gut dysfunction and fatigue. These symptoms correlate with markers of inflammation that persist well after the infection is cleared. In contrast, in endemic settings, first exposure occurs in children who are frequently malnourished and also co-infected with other enteropathogens. In these children, *Giardia* rarely causes symptoms and associates with several decreased markers of inflammation. Mechanisms underlying these disparate and potentially enduring outcomes following *Giardia* infection are not presently well understood. A body of work suggests that the outcome of experimental *Giardia* infection is influenced by the nutritional status of the host. Here, we explore the consequences of experimental *Giardia* infection under conditions of protein sufficiency or deficiency on cytokine responses of *ex vivo* bone marrow derived dendritic cells (BMDCs) to endotoxin stimulation. We show that BMDCs from *Giardia-* challenged mice on a protein sufficient diet produce more IL-23 when compared to uninfected controls whereas BMDCs from *Giardia* challenged mice fed a protein deficient diet do not. Further, *in vivo* co-infection with *Giardia* attenuates robust IL-23 responses in endotoxin-stimulated BMDCs from protein deficient mice harboring enteroaggregative *Escherichia coli*. These results suggest that intestinal *Giardia* infection may have extra-intestinal effects on BMDC inflammatory cytokine production in a diet dependent manner, and that *Giardia* may influence the severity of the innate immune response to other enteropathogens. This work supports recent findings that intestinal microbial exposure may have lasting influences on systemic inflammatory responses, and may provide better understanding of potential mechanisms of post-infectious sequelae and clinical variation during *Giardia* and enteropathogen co-infection.

## Author summary

*Giardia* is a leading cause of gut infection worldwide, with wide variation in acute symptoms. *Giardia* infection in non-endemic regions has also been associated with debilitating chronic intestinal and extra-intestinal sequelae, such as irritable bowel syndrome, often occurring weeks or months after exposure and after the parasite has cleared. Mechanisms underlying how *Giardia* results in an increased risk of chronic intestinal and extra-intestinal sequelae are not currently well understood. However, an emerging body of research suggests that intestinal microbial exposures may have long term influences on inflammatory cytokine production from innate immune cells, and that this process may help explain persistent inflammatory responses following pathogen infection. We therefore hypothesized that *Giardia* infection could alter innate immune system cells to be more pro-inflammatory. In this work we demonstrate that *Giardia* exposure in a murine model changes the inflammatory profile of bone marrow derived innate immune cells in in a diet dependent manner and in response to bacterial stimuli. These data suggest that *Giardia* infection might alter the responsiveness of innate immune cells to other infectious agents or natural products of the host microbiota such as endotoxin. This work may therefore provide better understanding of potential immune mechanisms underlying gut dysfunction following traveler’s diarrhea, as well as variation in disease severity in endemic regions.

## Introduction

*Giardia lamblia* is one of the most commonly reported intestinal parasitic infections worldwide [1–3]. Exposure results in a wide spectrum of outcomes, ranging from asymptomatic colonization, to acute or chronic diarrhea. Increasingly, post-infectious sequelae have been observed that include development of conditions that may be associated with altered inflammatory profiles [4,5] such as irritable bowel syndrome and chronic fatigue syndrome [6–11]. Mechanisms that might account for the broad range of clinical manifestations of giardiasis are poorly understood. However, there appear to be patterns of clinical and post-infectious outcomes that associate with global regions of high and low enteropathogen burden and malnutrition. *Giardia* infection outbreaks in adults in resource-abundant regions, including the USA [3] and Norway [10,12], increase risk for post-infectious irritable bowel syndrome (IBS) [3], duodenitis [10,11], persistent elevation of fecal calprotectin [12], and even lingering elevations in serum sCD14 [3,12]. In children living in resource-limited endemic settings with a high prevalence of recurrent and persistent infection, however, *Giardia* does not associate with diarrhea or routine markers of intestinal inflammation [2,13,14]. Rather, in these children who often have restricted dietary protein intake, *Giardia* associates with increased intestinal permeability, but decreased levels of myeloperoxidase (MPO). Also, in endemic settings, *Giardia* infection is associated with decreased levels of the serum inflammatory mediator CRP [2,13,15,16]. These diminished markers of inflammation associate with *Giardia* infection in endemic areas despite the presence of other potentially pro-inflammatory co-enteropathogen exposures such as enteroaggregative *Escherichia coli* (EAEC) [17].

We have shown that outcomes during persistent experimental *Giardia* infection in mice are diet dependent [18]. Chronically infected, fully nourished animals develop lymphocytic duodenitis similar to some chronic infections in adults in low endemicity settings. However, mice fed a protein deficient diet do not [17]. This diet-dependent immune discrepancy is opposite of the otherwise robust mucosal and systemic responses to *Cryptosporidium* or EAEC seen in this protein malnutrition model [17]. Further, *Giardia* challenge is sufficient to partially diminish calprotectin responses and MPO responses to EAEC [16–18]. There is an emerging body of research suggesting that previous intestinal exposures such as vaccination, alteration of the microbiota, or pathogen exposure, may have long term influences on systemic inflammatory cytokine production from innate immune cells [19]. We have previously shown that this process may help explain persistent inflammatory responses and disparate outcomes during pathogen infection and malnutrition [20–22].

Herein, we hypothesize that the unique immune profiles observed during persistent *Giardia* infection reflect *Giardia*-mediated alterations in innate, antigen presenting cell responses [23,24], and that host nutritional status influences these responses. *Ex vivo*, GM-CSF expanded bone marrow derived dendritic cells (BMDCs) represent a heterogeneous mix of terminally differentiated cells that can provide an *in vitro* model of characteristics of the innate antigen presenting cell population in an animal [25–27]. Therefore, we profiled the cytokine response of BMDCs from *Giardia* challenged mice on either a protein deficient or a protein sufficient control diet to explore how diet and *Giardia* infection might influence cytokine production following exposure to endotoxin. We also explored the relationship between *Giardia* colonization followed by co-infection with EAEC during malnutrition, as these co-infections are common in malnourished children in endemic settings.

## Results

**Fig 1:**
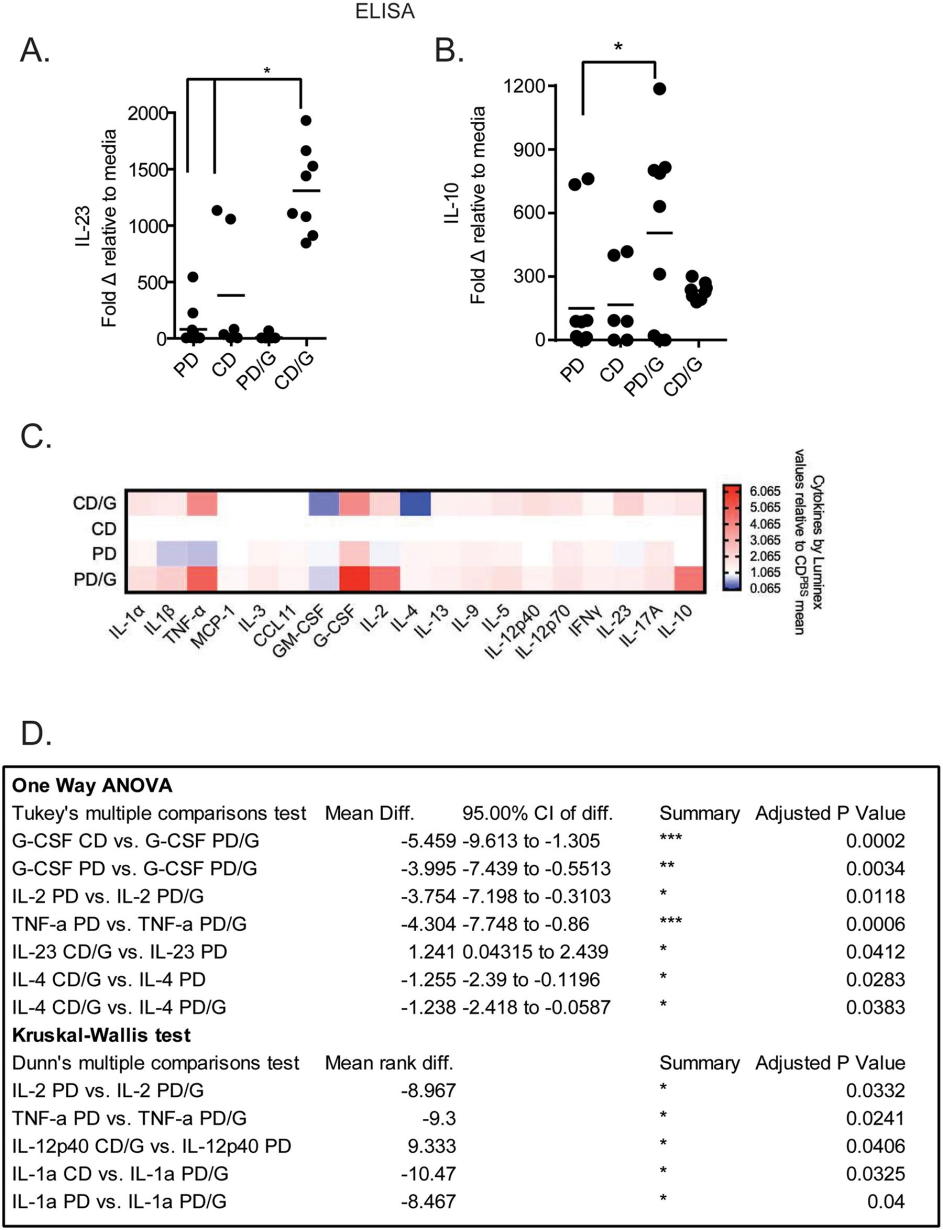
Cytokine production by BMDCs in response to endotoxin is altered during giardiasis in a diet dependent manner. Bone marrow cells were harvested from male C57BL/6 mice on a control (CD) or protein deficient diet (PD) that were challenged (CD/G, PD/G) or free of *Giardia lamblia* (CD, PD). Cells were cultured in RPMI with 10% FBS, supplemented on days 0 and 3 with GM-CSF (10ng/mL; Peprotech), and harvested on day 6. 2.5x10^5^ cells were plated per well of a round bottom 96-well dish, and treated with cell culture media or LPS for 24h. Cytokine levels in the supernatants were determined by ELISA (A, B) and Luminex (C, D). For ELISA data, values in (A, B) represent fold change of cytokines after LPS treatment (1mcg/mL) compared to unstimulated media control. * = p<0.05 One-Way ANOVA with Tukey post-test. For Luminex data, Cytokine values were normalized using fold-change relative to the mean value of the control group (C, D) for each cytokine and then compared using One-Way ANOVA, Kruskal-Wallis and Tukey or Dunn’s post-test (D), p-value is as reported in D. A,B N = 6–12 mice, C,D n = 3–6 mice.

In order to test how *Giardia* infection during protein deficiency and sufficiency influences cytokine production from *ex vivo* marrow cells we isolated bone marrow from infected and uninfected mice, expanded BMDCs, challenged with LPS, and measured cytokine production via ELISA and a multiplex Luminex assay. We show that BMDCs from *Giardia*-challenged mice produce more IL-23 in response to endotoxin than uninfected controls (Fig 1A, 1C and 1D) as measured via ELISA and Luminex. In contrast, BMDCs from *Giardia*-challenged mice fed a protein deficient (PD) diet do not exhibit this increase and produce more IL-10 as measured via ELISA (Fig 1B). *Giardia*-challenged mice on a protein deficient diet (PD) also produce more G-CSF, TNF-a, IL-1a and IL-2 than uninfected mice as measured by Luminex (Fig 1C and 1D). BMDCs from *Giardia* challenged mice on the protein sufficient control diet (CD) produce higher levels of IL-23, as mentioned above, as well as IL-12p40 (but decreased IL-4) as measured via Luminex (Fig 1C and 1D).

**Fig 2:**
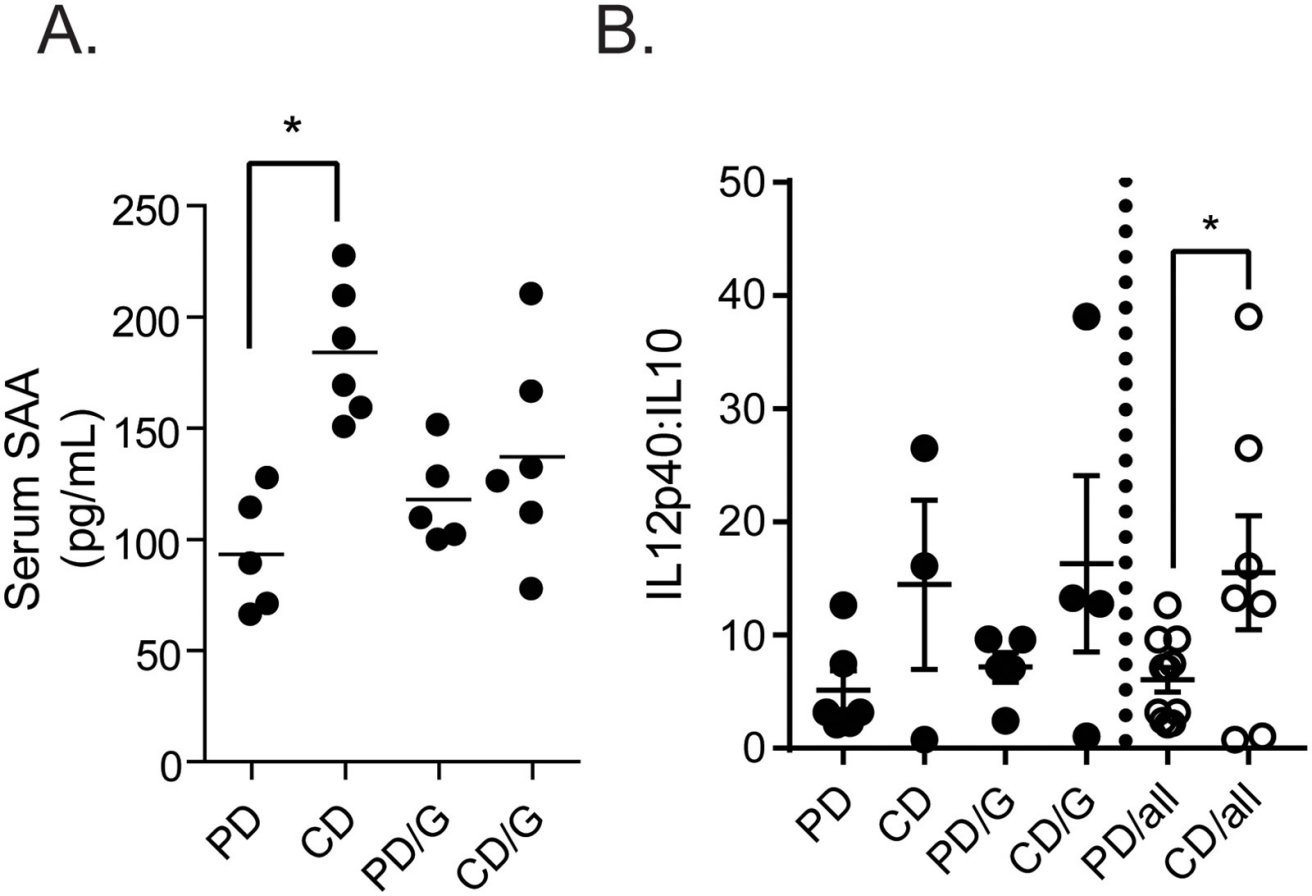
Serum SAA levels and IL-12p40: IL-10 ratios are altered by diet. Male C57BL/6 mice on a control (CD) or protein deficient diet (PD) were challenged (/G) or free of *Giardia lamblia* and serum level of SAA in each group was determined by ELISA (A). Bone marrow cells were harvested from male C57BL/6 mice on a control (CD) or protein deficient diet (PD) that were challenged (/G) or free of *Giardia lamblia*. Cells were cultured in RPMI with 10% FBS, supplemented on days 0 and 3 with GM-CSF (10ng/mL; Peprotech), and harvested on day 6. 2.5x10^5^ cells were plated per well of a round bottom 96-well dish, and treated with cell culture media or LPS for 24h. Cytokine levels in the supernatants were determined by ELISA (B) * = p<0.05 One-Way ANOVA, Tukey posttest. n = 5–6 mice.

Previous studies have suggested that serum mediators, such as the damage associated molecular pattern molecule serum amyloid A (SAA) produced by the host, may increase IL-23 production from *ex vivo* LPS stimulated BMDCs [28]. In these experiments, uninfected mice fed the control diet (CD) have significantly higher levels of serum SAA than those fed the protein deficient (PD) diet (Fig 2A). Macrophages and myeloid derived antigen presenting cells (APCs), which BMDCs partially model, can be differentially polarized towards a Th1 or Th2 cytokine producing phenotype, with a continuum of phenotypes in between [29]. Therefore, we utilized the IL-12p40:IL-10 ratio as a marker of potential dendritic cell/macrophage polarity [30,31], and found reduced IL-12p40:IL-10 ratios in LPS-stimulated BMDCs from mice fed the PD diet (Fig 2B).

**Fig 3:**
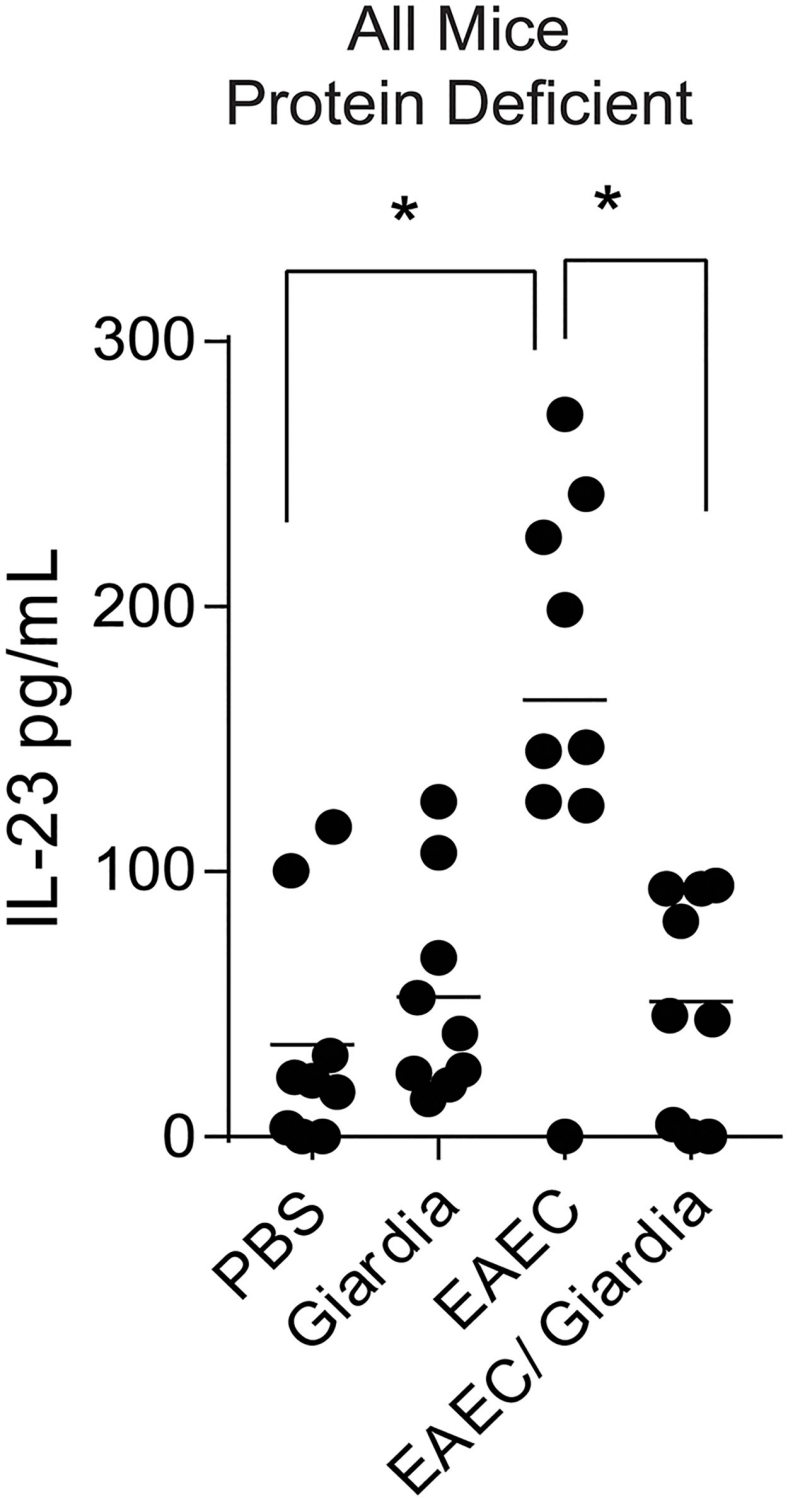
*Giardia* co-infection during protein deficiency and *Enteroaggregative Escherichia coli* (EAEC) infection suppresses increased IL-23 production by EAEC. Bone marrow cells were harvested from male C57BL/6 mice that were on a protein deficient diet (PD) upon arrival at 3 weeks of age and then challenged with *G*. *lamblia* 7 days later. Six days after *G*. *lamblia* exposure mice were challenged with enteroaggregative *E*. *coli* (EAEC) and euthanized on day 21 following *Giardia* infection. Cells were cultured in RPMI with 10% FBS, supplemented on days 0 and 3 with GM-CSF (10ng/mL; Peprotech), and harvested on day 6. 2.5x10^5^ cells were plated per well of a round bottom 96-well dish, and treated with cell culture media or LPS for 24h. Cytokine levels in the supernatants were determined by ELISA (B) * = p<0.05 One-Way ANOVA, Tukey posttest. n = 9 mice.

Most *Giardia* infections in endemic settings occur sequentially or simultaneous with other co-pathogens, and *Giardia* is frequently detected together with enteric bacteria [2]. Thus, we used our co-infection model system [17] in PD-fed mice to determine whether prior *Giardia* infection would alter cytokine production from BMDCs after subsequent co-infection with EAEC. BMDCs were cultured from mice on the PD diet infected with *Giardia* alone, EAEC alone, or *Giardia* and then EAEC. Unlike *Giardia* alone, EAEC alone significantly increased IL-23 in response to LPS (Fig 3). However, prior *Giardia* colonization attenuated IL-23 responses in BMDCs from EAEC infected mice.

## Discussion

Previous studies have suggested that *Giardia* may exacerbate colitis in genetically pre-disposed hosts (*IL10-/-* mice) through increased IL12p40/IL-23 production [32]. Our results suggest that *Giardia* infection led to extra-intestinal effects on BMDC inflammatory cytokine responses to LPS stimulation in a diet-dependent manner. Precise mechanisms underlying these differences in cytokine profile are not yet understood. Intestinal infection with one organism, or vaccination [21], may persistently alter innate immune populations to provide a more robust response to infection with unrelated pathogens in a process coined “*trained immunity*” [19,33]. The mechanism of trained immunity is not well understood, but may epigenetic changes in genes important in innate immunity have been implicated [34–37]. These changes might include methylation of H3K27 and H3K4 histones associated with promotor regions of multiple genes, including, but not limited to, IL-23 [36].

Epigenetic effects may underlie the phenotype observed in our data, and this will be examined in future studies. In our experiments, mice on a control diet (CD) have significantly higher levels of SAA than those fed the protein deficient (PD diet). Disparate serum levels of host derived damage associated molecular pattern molecules (DAMPs) such as the acute phase protein serum amyloid A (SAA), are induced by the microbiota or infection and have been shown to be important in upregulating expression of epigenetic mediators such as histone demethylases and IL-23 in both myeloid cell lines and bone marrow [28,36,38–40]. SAA treatment preferentially increases secretion of IL-23, but not IL-12, in peripheral blood monocytes and the THP-1 monocytic cell line [40]. Increased serum levels of SAA, and direct culture with SAA, has been associated with increased IL-23 production in murine dendritic cells [28]. Thus, the decreased SAA during protein deficiency could limit IL-23 production from BMDCs following *Giardia* infection. Interestingly, decreased serum SAA associates with linear growth restriction early in life in endemic areas, but as enteric infection exposures like *Giardia* accumulate, elevated SAA predicts poorer subsequent growth [41].

Altered serum SAA may not fully explain our result as other metabolites or mediators may play a significant role in modulating cytokine production. Indeed, we observed increased IL-10 production by BMDCs from *Giardia*-infected mice fed the protein deficient diet. Furthermore, using the IL-12p40:IL-10 ratio as a marker of potential dendritic cell/macrophage polarity [30,31], we found reduced IL-12p40:IL-10 ratios in LPS-stimulated BMDCs from mice fed the PD diet. These data suggest that the combination of *Giardia* and protein deficiency leads to an altered inflammatory environment. This in turn, might influence the inflammatory profile of innate immune cells during co-infection with another enteropathogen. Thus, to determine whether harboring *Giardia* during protein deficiency altered BMDC responses following EAEC co-infection, BMDCs from mice infected with *Giardia* alone, EAEC alone, or *Giardia* and then EAEC or were stimulated with LPS. Again, *Giardia* alone did not significantly increase IL-23 in these protein deficient mice, whereas EAEC alone did. Prior *Giardia* colonization, however, resulted in a minimal IL-23 response to EAEC. These data are consistent with our previous finding that *Giardia* alters intestinal calprotectin production during EAEC co-infection, and points to *Giardia*-mediated changes in myeloid cell activation [17]. IL-10 signaling during *Giardia* infection has recently been shown to be important in preventing development of colitis in mice [32]. Interestingly, our results suggest that during malnutrition *Giardia* infection may induce innate IL-10 production, that might partially attenuate inflammatory responses induced by enteropathogens that cause enterocolitis.

**Fig 4:**
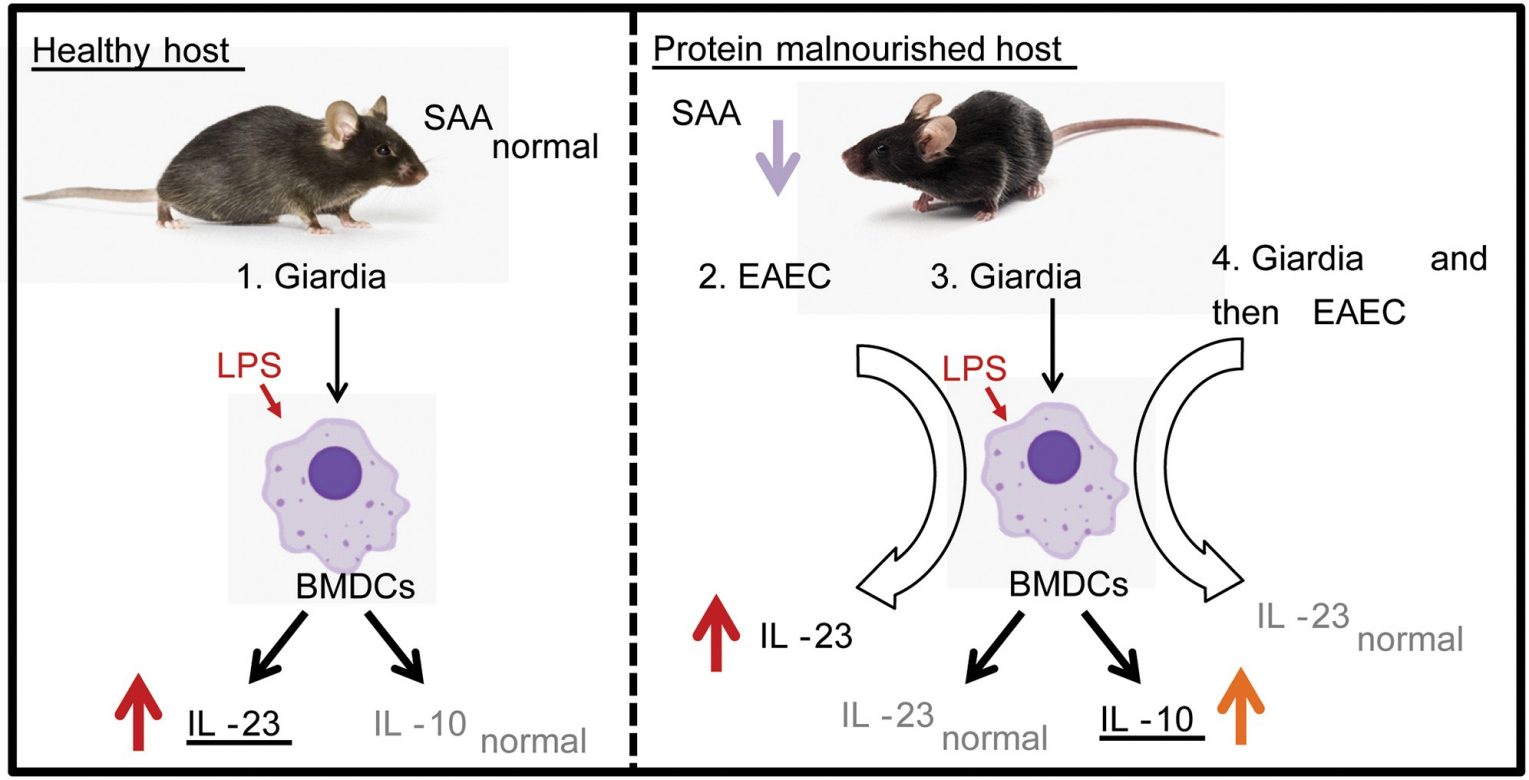
Diet-dependent changes in bone marrow derived dendritic cell inflammatory cytokine production occur during both *Giardia* and *Giardia* and enteroaggregative *E*. *coli* (EAEC) co-infection. We observed increased inflammatory cytokine production (IL-23) from bone marrow derived DCs isolated from *Giardia* Infected mice when the mice were on a protein sufficient diet compared to uninfected mice **(1)**. EAEC infected mice on a protein deficient diet also produced more IL-23 **(2)**. BMDCs derived from *Giardia* infected mice on a protein deficient diet produced more IL-10 when compared to uninfected mice however **(3)**. The increase in IL-23 observed during EAEC infection was lost when the mice were infected with *Giardia* prior to EAEC **(4)**. This data suggests that *Giardia* infection during protein sufficiency may alter the inflammatory profile of bone marrow derived cells. Conversely, the parasite may attenuate increased IL-23 production from marrow derived cells during malnutrition and coinfection with other enteric pathogens.

Previous *in vitro* co-culture experiments have demonstrated that *Giardia* can directly modulate cytokine production from dendritic cells, with disparate outcomes depending on culture conditions, agonists, and cell type [9,42]. To our knowledge this is the first demonstration that a non-invasive intestinal protozoa alters systemic cytokine profiles of BMDCs and in a diet-dependent manner. Our work suggests that *Giardia* infection during, or in the absence of protein malnutrition, might persistently alter the responsiveness of innate immune cells to later challenge with infectious agents or their products, as measured via cytokine production (Fig 4). Limitations of the work involve the model system of innate bone marrow immune derived cells (BMDCs). Thus, we were unable to define the specific cell within the BMDC culture responsible for the functional response. Future studies will examine the influence of *Giardia* infection on proliferation, trafficking and inflammatory potential of hematopoietic and distinct intestinal myeloid immune populations [39]. *Giardia* infection may also influence hematopoietic cells, as previous studies have suggested that changes in the cytokine responsiveness of BMDCs mediated by the microbiota also correlate with changes in myeloid precursor cells in the marrow [28,39]. This work is beyond the scope of the current investigation however. In conclusion, we demonstrate that intestinal *Giardia* infection has extra-intestinal effects on BMDC inflammatory cytokine production in a diet dependent manner. Furthermore, *Giardia* infection during protein deficiency and co-infection attenuates innate IL-23 responses elicited by the co-infecting enteropathogen (EAEC). Ultimately these studies may help provide better understanding of potential immune mechanisms underlying a range of post-infectious gut dysfunction, environmental enteric dysfunction [43] and other extra-intestinal sequelae due to enteropathogen infection, as well as clinical variability.

## Materials and methods

### *Giardia lamblia* and enteroaggregative *Escherichia coli* preparations

Gerbil-passaged purified *G*. *lamblia* H3 (Assemblage B) cysts were purchased from Waterborne, Inc. (New Orleans, LA). Cysts were washed and diluted in PBS and used within 48 hours of arrival. The EAEC strain 042 was originally obtained from James Nataro at the University of Virginia. For each experiment, a separate inoculum of 10^9^/mouse was grown from a glycerol stocked maintained at -80°C and prepared in DMEM high glucose medium. All pathogen preparations were maintained on ice until administered via oral gavage using 22-gauged feeding needles in 100 *μ*L volumes. Uninfected controls were similarly gavaged with either 100 *μ*L of PBS (for *Giardia*) or DMEM high glucose (for EAEC) control.

#### Animals, diets, and infection

All experiments were performed using weaned male C57BL/6 mice received from Jackson Laboratories at 3 weeks of age. Mice were initiated on either a protein deficient diet (PD; 2% protein, Teklad, Envigo) or an isocaloric control diet (CD; 20% protein, Teklad, Envigo) upon arrival. Mice were randomized into weight-matched groups and continued on experimental diets ad libitum throughout the duration of the experiment. Mice were challenged with 10^7^ purified *G*. *lamblia* cysts (Assemblage B, H3, Waterborne, Inc) after 21 days on diet (day of life 42). For EAEC experiments C57BL/6 males were initiated on the protein deficient diet upon arrival at 3 weeks of age and then challenged with *G*. *lamblia* 7 days later. Six days after *G*. *lamblia* mice were challenged with enteroaggregative *E*. *coli* (EAEC). Uninfected controls received PBS-PBS sham gavages at each infection timepoint. Mice were euthanized and tissues were harvested in two batches consisting of equal numbers of mice from each of the four experimental groups, on either day 20 or 21 after *Giardia* challenge. N = 3–12 mice per group were utilized for all experiments and all data is included in S1 Data.

#### Bone marrow derived dendritic cell (BMDC) culture

Bone marrow cells were harvested and immediately cryopreserved on-site at UNC-CH according to prior published methods [28]. Cryopreserved cells were transported on dry ice to the University of Virginia and stored at -80 °C. Cells were thawed and cultured in RPMI with 10% FBS supplemented on days 0 and 3 with GM-CSF (10 ng/mL; Peprotech), and harvested on day 6. For *in vitro* experiments, 2.5x10^5^ cells were plated per well of a round-bottom 96-well dish, and treated with lipopolysaccharide (Ultra-pure LPS-EK, Invitrogen, 1mcg/mL) for 24h. Cytokines in the supernatants were determined by ELISA (IL-23, IL-10 R&D Systems) and Luminex (DropArray 96 plate for Bio-PlexTM, Curiox). Cells from at least three mice per group, in duplicate, were utilized. Results were analyzed using One Way ANOVA or, Kruskal-Wallis test with Turkey or Dunn’s post -test, based on Shapiro–Wilk test for normality, in Graphpad Prism 7 software (San Diego, CA).

**Serum SAA** was measured by ELISA (Abcam, Cambridge, England, ab157723).

### Ethics statement

All experiments and procedures were approved by the Institutional Animal Care and Use Committee of the University of North Carolina at Chapel Hill (IACUC # 15–345). All experiments were performed according to provisions of the USA Animal Welfare Act of 1996 (Public Law 89.544).

## Supporting information

S1 Data All data presented in this manuscript is available in digital format in an excel xlsx worksheet. (XLSX)
